# A retrospective real-world study: the efficacy of immune-related combination therapies in advanced non-small cell lung cancer after resistance to EGFR-TKIs

**DOI:** 10.1007/s00262-023-03570-9

**Published:** 2023-10-31

**Authors:** Ruoxue Cai, Ying Liu, Mingyan Yu, Huanhuan Sha, Mengya Guo, Yue Chen, Jinjun Ye, Guoren Zhou, Ying Fang, Bo Shen

**Affiliations:** 1 Department of Oncology, The Affiliated Cancer Hospital of Nanjing Medical University, Jiangsu Cancer Hospital, Jiangsu Institute of Cancer Research, Baiziting 42, 210009 Nanjing, People’s Republic of China; 2 Department of Radiotherapy, The Affiliated Cancer Hospital of Nanjing Medical University, Jiangsu Cancer Hospital, Jiangsu Institute of Cancer Research, 210009 Nanjing, People’s Republic of China; 3 Department of Oncology, Jiangsu Cancer Hospital, Jiangsu Institute of Cancer Research, The Affiliated Cancer Hospital of Nanjing Medical University, 210009 Nanjing, People’s Republic of China

**Keywords:** Immune checkpoint inhibitors (ICIs), Epidermal growth factor receptor-tyrosine kinase inhibitors (EGFR-TKIs) resistance, Non-small cell lung cancer (NSCLC), Brain metastasis, Liver metastasis

## Abstract

**Background:**

Whether patients with advanced non-small cell lung cancer (NSCLC) should choose an immune-combination therapy regimen after EGFR-tyrosine kinase inhibitors (EGFR-TKIs) resistance is currently unclear.

**Methods:**

We evaluated 118 NSCLC patients treated by immune checkpoint inhibitors (ICIs) + chemotherapy (I + C), ICIs + chemotherapy + antiangiogenic therapy (I + C + A), chemotherapy + antiangiogenic therapy (C + A) after inefficacy of EGFR-TKIs. We assessed the objective remission rate (ORR), disease control rate (DCR), and progression-free survival (PFS) of these treatments.

**Results:**

The ORR was 26.1% vs 38.2% vs 16.3% in the three groups (*P* = 0.093). The divergence in DCR was also statistically significant (65.2% vs 85.3% vs 74.4%, *P* = 0.209). The median PFS was no statistically significant difference in PFS (3.09 vs 6.31 vs 5.91 months, *P* = 0.809), but the Kaplan–Meier survival curve of 12-month-PFS indicated an apparent survival advantage in the I + C + A group (*P* = 0.001). In addition, the I + C/I + C + A group showed higher median PFS than the C + A group in patients with brain metastases (median PFS, 6.44 vs 4.21 months, *P* = 0.022). The divergence in ORR of patients in the brain group was also statistically significant (*P* = 0.045). The I + C + A group showed superior efficacy in patients with liver metastases (median PFS, 0.95 vs 6.44 vs 3.48 months, *P* < 0.0001). The Cox proportional hazard modeling analysis suggested that the age, brain metastases, and liver metastases were all connected with the prognosis.

**Conclusions:**

This study suggests that advanced NSCLC patients after resistance to EGFR-TKIs may achieve better outcomes from triple therapy. Patients with brain metastases favor ICIs-related combination therapies and patients with liver metastases prefer I + C + A therapy.

**Supplementary Information:**

The online version contains supplementary material available at 10.1007/s00262-023-03570-9.

## Introduction

Lung cancer is a kind of primary cancer with high mortality and incidence, non-small cell lung cancer (NSCLC) is the most common type of lung cancer [1]. The occurrence of epidermal growth factor receptor (EGFR) mutation and the emergence of EGFR-tyrosine kinase inhibitors (EGFR-TKIs) have changed the treatment prospect of NSCLC, and EGFR-TKIs are now commonly used in the first or subsequent lines of therapy for NSCLC patients with EGFR mutations [2]. Nevertheless, although the third-generation EGFR-TKIs Osimertinib overcame the resistance to the T790M mutation, with the emergence of other mechanisms of resistance in tumor cells, such as MET amplification [3], Her2 amplification [4], C797X mutations [5], and histological transformations [6], the third-generation TKIs were not immune to the fate of drug resistance. Currently, these resistant patients are generally treated with platinum-containing two-agent chemotherapy with or without bevacizumab [7, 8]. Although studies have shown that chemotherapy in combination with bevacizumab results in a better prognosis than chemotherapy alone [9, 10], chemotherapy in combination with bevacizumab provides minimal survival benefit for patients after EGFR-TKIs resistance. Therefore, new therapeutic approaches for patients who do not develop T790M mutation after a former application of first-generation or second-generation EGFR-TKIs resistance and for patients who remain resistant after the application of third-generation EGFR-TKIs therapy following the appearance of T790M mutation still need to be further explored.

For the past few years, the treatment prospect of NSCLC has been revolutionized because of the discovery of immune checkpoint inhibitors (ICIs). Multiple large studies demonstrated that ICIs significantly improve survival prognosis in advanced NSCLC patients without EGFR mutations [11–13]. The research indicated that NSCLC patients with positive PD-L1 expression but without EGFR mutations achieved better progression-free survival (PFS) and overall survival (OS) with pembrolizumab in comparison to chemotherapy [14]. However, immunologic monotherapy has limited benefits in NSCLC patients with EGFR mutations. Immunologic monotherapy has been shown to be ineffective in PD-L1 positive NSCLC patients with EGFR mutations after EGFR-TKIs resistance [15].

Notably, there is a mutual synergy between ICIs and chemotherapy, with chemotherapy inducing PD-L1 expression, removing suppressor immune cells, and enhancing effector T-cell functions [16, 17]. Antiangiogenic drugs combined with ICIs can enhance antitumor immune effects by improving PD-L1 expression, T-cell infiltration, and promoting vascular normalization in recurrent tumors [18, 19].

Based on the above mechanisms, an increasing number of studies such as the Impower150 trial, the Orient-31 trial et.al focused on the efficacy of immune-combination therapies in NSCLC patients [20, 21]. However, no definitive studies showed which combination therapy between immunotherapy, anti-angiogenic therapy and chemotherapy is the most efficacious strategy in EGFR-mutated NSCLC patients after EGFR-TKIs resistance.

What’s more, distance metastasis has long been another challenge to the survival of advanced NSCLC patients. Although third-generation EGFR-TKIs Almonertinib approved in China was effective in improving ORR and DCR of brain metastatic lesions in EGFR-mutated NSCLC patients [22], what kind of treatment options should be chosen after its resistance is still worth further exploration.

In our retrospective study, we compared the efficacy of three combination therapies to investigate which combination therapy provides a better survival benefit for NSCLC patients after EGFR-TKIs resistance, especially in the presence of brain and liver metastasis.

## Methods

### Study population

This is a retrospective clinical study, we retrospectively collected clinical data from electronic medical records of NSCLC patients from June 1, 2019, to May 1, 2023, at Jiangsu Cancer Hospital. NSCLC patients with EGFR mutations who were treated by ICIs combined with chemotherapy (I + C), ICIs combined with anti-angiogenic therapy and chemotherapy (I + C + A), and chemotherapy combined with anti-angiogenic therapy (C + A) after resistance to EGFR-TKIs were analyzed. These patients have pathologically confirmed NSCLC and the existence of EGFR mutations was detected by next-generation sequencing, they did not show histologic transformation of small-cell lung cancer. Before receiving treatment, they had to have previously received the treatment of first-generation or/and second-generation EGFR-TKIs followed by resistance and without T790M mutation, or the presence of T790M mutation but disease progression despite third-generation EGFR-TKIs requiring an alternative systemic therapy regimen. The Ethics Committee of the Affiliated Cancer Hospital of Nanjing Medical University has approved this study (NO. 2023–044).

### Research endpoints

The clinical data were gathered and analyzed based on the Response Evaluation Criteria in Solid Tumors version 1.1 (RECIST v1.1). Therapeutic responses included complete response (CR), partial response (PR), stable disease (SD), and progressive disease (PD). The proportion of patients who had a CR and PR of any metastasis (including the brain) was defined as the overall response rate (ORR). The proportion of patients who had a CR and PR and SD was defined as the disease control rate (DCR). PFS was defined as the initiation of treatment until disease progression. The OS was not assessed in this study because the majority of patients in the triple regimen group were newly enrolled and the majority of patients subsequently received other treatment regimens, making OS results potentially biased. The time was also cut off at the date of the last treatment if patients ceased therapy on account of non-progressive disease causes such as adverse reactions. In addition, we performed specialized imaging evaluations of brain and liver lesions. We defined the intracranial or intrahepatic progression-free survival (PFS) as the date of the start of ICIs therapy to the date of intracranial or intrahepatic lesions progressed. Patients who had extracranial or extrahepatic progression were not included in the analysis of intracranial PFS.

### Statistical analysis

We summarized clinical characteristics and adverse events data by descriptive statistical analysis. Analysis of PFS using the Kaplan–Meier methodology. We also analyzed survival in subgroups of patients with common distant metastasis. Using the log-rank test to contrast treatment efficacy between different groups. Univariate and multivariate analyses by Cox proportional hazard modeling were conducted to evaluate predictors associated with PFS. *P* < 0.05 were considered to be statistically significant. Variables in univariate analysis with *P* < 0.1 were chosen for multifactor analysis. We used SPSS Statistics 26 for data analysis and Graphpad Prism 9.5 for plotting.

## Results

### Patient characteristics

We finally screened 118 NSCLC patients with EGFR mutations from The Affiliated Cancer Hospital of Nanjing Medical University who received one of the three combination therapies after EGFR-TKIs resistance. To exclude factors that might affect prognosis, we performed propensity score matching analysis for age, number of lines of treatment, genetic test results, and whether or not to combine radiotherapy. Finally, 100 patients were included in the efficacy comparison.

Most of the patients were aged < 65 years (63.00%), and with a histologic diagnosis of adenocarcinoma (94.00%), denied smoking history (84.00%), the Eastern Cooperative Oncology Group (ECOG) performance status was 0–1 (73.00%) and had no combined with local radiotherapy (91.00%). Brain metastasis appeared in 55 patients (55.00%), liver metastasis in 29 patients (29.00%), bone metastasis in 56 patients (56.00%), and adrenal metastasis in 17 patients (17.00%). All patients had EGFR mutations, but concrete clinical information from genetic testing was obtained for a total of 92 patients. Primary EGFR mutations included 43 EGFR 19del (43.00%), 49 EGFR L858R (49.00%), 1 EGFR S768I and 1 EGFR L861Q. 57 patients have been treated with third-generation EGFR-TKIs (57.00%). A total of 57 (57.00%) patients received immune-related combination therapy, of which 23 received immune-combination chemotherapy (I + C), and 34 received immunotherapy + chemotherapy + antiangiogenic therapy (I + C + A). 43 patients (43.00%) received chemotherapy in combination with antiangiogenic therapy (C + A). The specified clinical characteristics of all patients after propensity score matching analysis are summarized below. (Table 1) (Supplemental Table 1).

**Table 1:** Clinical characteristics of the study population after propensity score matching analysis

Characteristic	N (%)			
	All (N = 100)	I + C (N = 23)	I + C + A (N = 34)	C + A (N = 43)
*Age at the start of treatment*				
≥ 65	37 (37.00)	10(43.38)	11 (32.35)	16 (37.21)
< 65	63 (63.00)	13 (56.52)	23 (67.65)	27 (62.79)
*Gender*				
Male	45 (45.00)	11(47.83)	16 (47.06)	18 (41.86)
Female	55 (55.00)	12 (52.17)	18 (52.94)	25 (58.14)
*Smoking history*				
Never	84 (84.00)	19 (82.61)	27 (79.41)	38 (88.37)
Smoked	16 (16.00)	4 (17.39)	7 (20.59)	5 (11.63)
*ECOG score*				
0–1	73 (73.00)	17 (73.91)	21 (61.76)	35 (81.40)
≥ 2	27 (27.00)	6 (26.09)	13 (38.24)	8 (18.60)
*Pathologic type*				
Adenocarcinoma	94 (94.00)	20 (86.96)	32 (94.11)	42 (97.67)
Squamous carcinoma	4 (4.00)	3(13.04)	0 (0.00)	1 (2.33)
Mixed type	2 (2.00)	0 (0.00)	2 (5.89)	0 (0.00)
*Sites of metastasis*				
Brain	55 (55.00)	11 (47.83)	21 (61.76)	23 (53.49)
Liver	29 (29.00)	4 (17.39)	11 (32.35)	14 (32.56)
Bone	56 (56.00)	12 (52.17)	18 (52.94)	26 (60.46)
Adrenal gland	17 (17.00)	5 (21.74)	6 (17.65)	6 (13.95)
*Primary EGFR mutation*				
EGFR 19del	43 (43.00)	9 (39.13)	19 (55.88)	15 (34.88)
EGFR 21L858R	49 (49.00)	12 (52.17)	12 (35.30)	25 (58.14)
others	2 (2.00)	2 (8.70)	3 (8.82)	3 (6.98)
*Acquired T790M mutation*				
Yes	27 (27.00)	6 (26.09)	9 (26.47)	12 (27.91)
No or unknown	73 (73.00)	17 (73.91)	25 (73.53)	31 (72.09)
*Previous EGFR-TKIs treatment*				
1st/2nd generation TKI	43 (43.00)	14 (60.87)	10 (29.41)	25 (58.14)
1st/2nd➝3rd generation TKI	41 (41.00)	6 (26.09)	17 (50.00)	12 (27.91)
3rd generation TKI	16 (16.00)	3 (13.04)	7 (20.59)	6 (13.95)
*Prior lines of therapy*				
≤ 2	25 (25.00)	2 (8.70)	12 (35.29)	11 (25.58)
> 2	75 (75.00)	21 (91.30)	22 (64.71)	32 (74.42)
*Concurrent radiotherapy*				
No	91 (91.00)	20 (86.96)	32 (94.12)	39 (90.70)
Yes	9 (9.00)	3 (13.04)	2 (5.88)	4 (9.30)
*Radiotherapy site*				
Lung	6 (66.67)	2 (66.67)	2 (100.00)	2 (50.00)
Bone	3 (33.33)	1 (33.33)	0 (0.00)	2 (50.00)
Brain	0 (0.00)	0 (0.00)	0 (0.00)	0 (0.00)

*ECOG* Eastern cooperative oncology group; *EGFR* Epidermal growth factor receptor; *19del* Exon 19 deletion; *21L858R* Exon 21 L858R mutation; *TKI* Tyrosine kinase inhibitor; *I* + *C* Immunotherapy + chemotherapy combination treatment; *I* + *C* + *A* Immunotherapy + chemotherapy + antiangiogenic combination treatment; *C* + *A* Chemotherapy + antiangiogenic combination treatment

### Assessment of efficacy

**Fig. 1:**
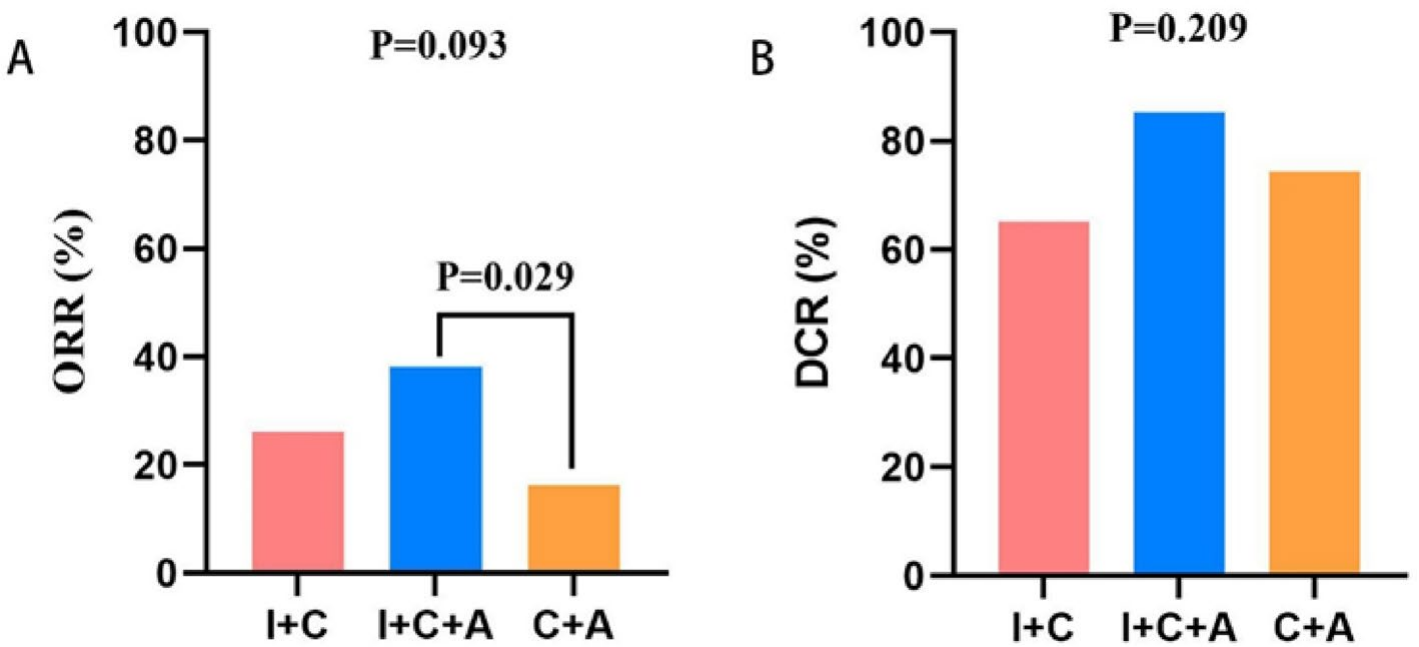
Comparison of ORR and DCR of all patients in three groups: **A** the overall response rate (ORR). **B** the disease control rate (DCR)

Imaging data were available for 100 patients for specific efficacy assessment, with 3 patients achieving CR and 24 patients achieving PR, for an overall ORR of 26.0% and an overall DCR of 76.0%. The highest ORR and DCR were found in the I + C + A group, which were 38.2% and 85.3%, respectively. However, a statistically significant difference was not found between these three groups when comparing the ORR (*P* = 0.093) and DCR (*P* = 0.209). The two-by-two comparison of the ORR and DCR of these three groups revealed that the difference between the I + C + A and C + A groups in the ORR (*P* = 0.029) was statistically significant. (Table 2) (Fig. 1).

**Table 2:** Treatment response in all patients

Patients	Response—N (%)				ORR	DCR
	CR	PR	SD	PD		
Total patients (n = 100)	3(3.0)	24(24.0)	51(51.0)	22(22.0)	26.0%	76.0%
I + C (n = 23)	0(0.0)	7(30.4)	8(34.8)	8(34.8)	26.1%	65.2%
I + C + A (n = 34)	2(5.9)	12(35.3)	17(50.0)	3(8.8)	38.2%	85.3%
C + A (n = 43)	1(2.3)	5(11.6)	26(60.5)	11(25.6)	16.3%	74.4%
*P*					0.093	0.209
I + C vs I + C + A					0.340	0.076
I + C vs C + A					0.340	0.431
I + C + A vs C + A					0.029	0.243

Objective response rate (ORR) = Complete response (CR) + Partial response (PR)

Disease control rate (DCR) = Complete response (CR) + Partial response (PR) + Stable disease (SD)

*I* + *C* Immunotherapy + chemotherapy combination treatment; *I* + *C* + *A* Immunotherapy + chemotherapy + antiangiogenic combination treatment; *C* + *A* chemotherapy + antiangiogenic combination treatment

**Fig. 2:**
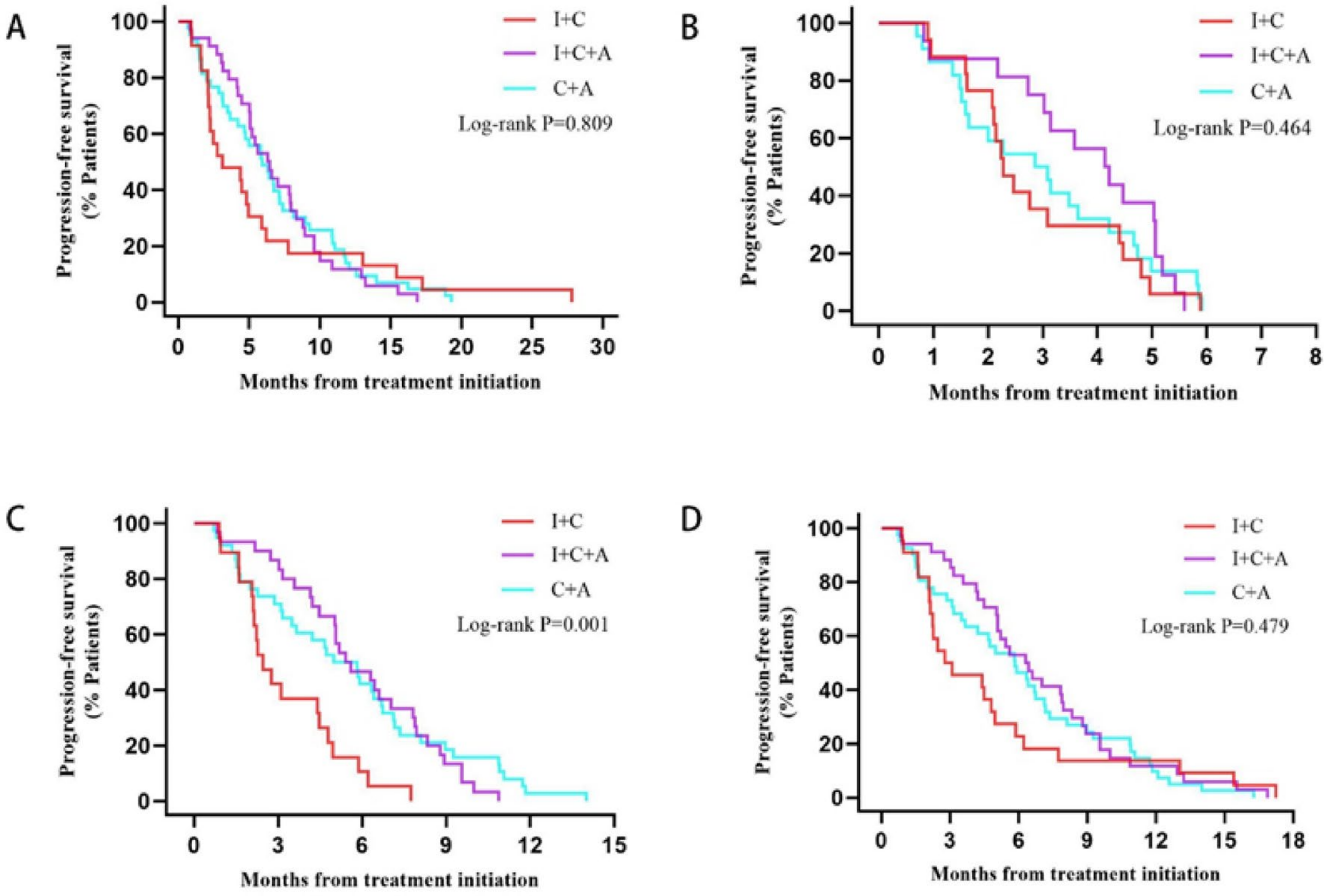
Kaplan–Meier estimates of progression-free survival (PFS) among the three groups: **A** the total PFS. **B** the 6-month-PFS, **C** the 12-month-PFS, **D** the 18-month-PFS

The median PFS was 5.42 months in all patients (95% CI 4.518–6.322), with a median PFS of 3.09 months (95% CI 0.054–6.126) in the I + C group, 6.31 months (95% CI 4.624–7.996) in the I + C + A group, 5.91 months (95% CI 4.085–7.735) in the C + A group. The I + C + A group tended to achieve longer PFS, although these three groups were without statistically significant differences (*P* = 0.809). Significantly, survival analysis of the PFS of the three groups at 6, 12, and 18 months found that the I + C + A group showed a notably higher 12-month PFS than the other two groups (*P* = 0.001), but there was no significant advantage in 6-month PFS and 18-month PFS. (Fig. 2).

### Evaluation of efficacy in patients with brain metastases

**Fig. 3:**
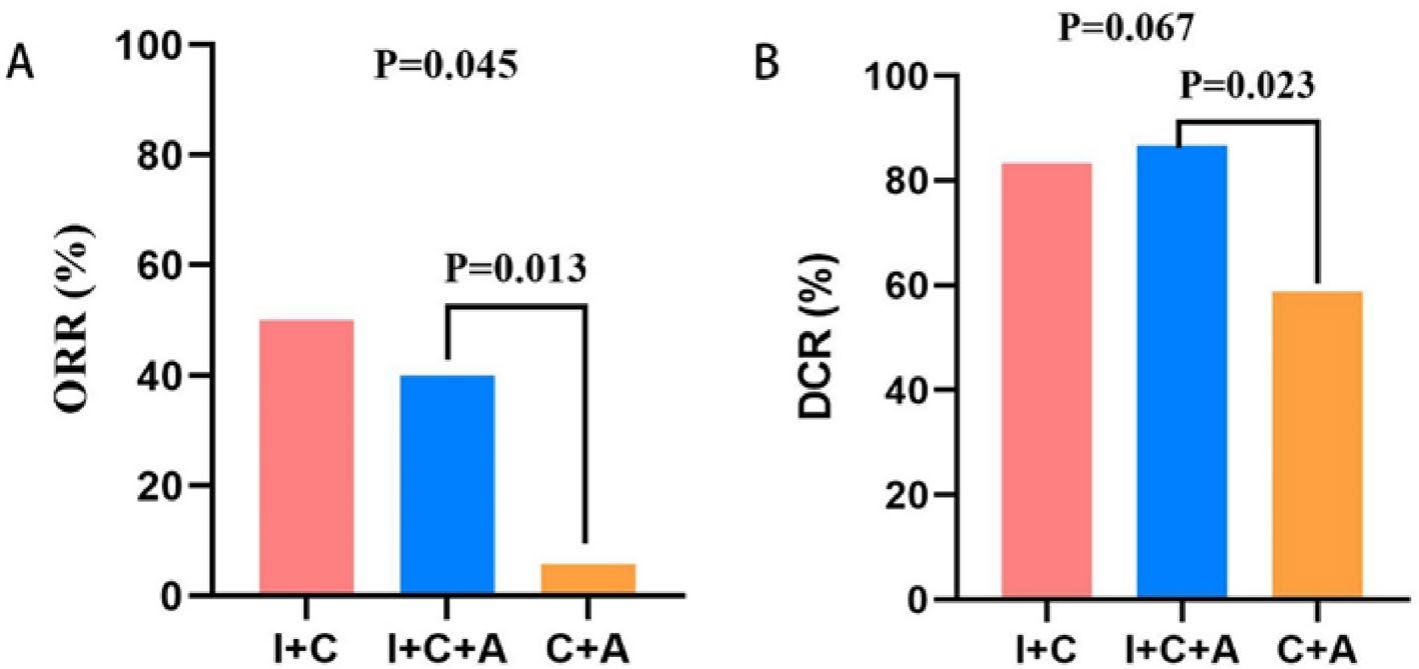
Comparison of ORR and DCR of all patients in the brain metastases group: **A** the overall response rate (ORR). **B** the disease control rate (DCR)

After we evaluated the brain metastatic lesions in 38 of the 55 patients with brain metastases among which imaging information was available, we found that the ORR was 50.0% in the I + C group, 40.0% in the I + C + A group, and 5.9% in the C + A group. There was statistical difference in the comparison between the three groups (*P* = 0.045), the ORR in the C + A group was significantly lower than the other two groups. In addition, the DCR was 83.3% in the I + C group, 86.7% in the I + C + A group, and 58.8% in the C + A group; Although there was also no statistically significant difference among the three groups (*P* = 0.067), the DCR in I + C and I + C + A group were apparently better than which in C + A groups. (Fig. 3) (Table 3).

**Table 3:** Treatment response in the brain metastases group

Patients	Response—N (%)				ORR	DCR
	CR	PR	SD	PD		
Total patients (n = 38)	3(7.9)	7(18.4)	18(47.4)	10(26.3)	26.3%	73.7%
I + C (n = 6)	1(16.7)	2(33.3)	2(33.3)	1(16.7)	50.0%	83.3%
I + C + A (n = 15)	2(13.3)	4(26.7)	7(46.7)	2(13.3)	40.0%	86.7%
C + A (n = 17)	0(0.00)	1(5.9)	9(52.9)	7(41.2)	5.9%	58.8%
P					0.045	0.067
I + C vs I + C + A					0.725	0.115
I + C vs C + A					0.052	0.714
I + C + A vs C + A					0.013	0.023

Objective response rate (ORR) = Complete response (CR) + Partial response (PR)

Disease control rate (DCR) = Complete response (CR) + Partial response (PR) + Stable disease (SD)

*I* + *C* Immunotherapy + chemotherapy combination treatment; *I* + *C* + *A* Immunotherapy + chemotherapy + antiangiogenic combination treatment; *C* + *A* chemotherapy + antiangiogenic combination treatment

**Fig. 4:**
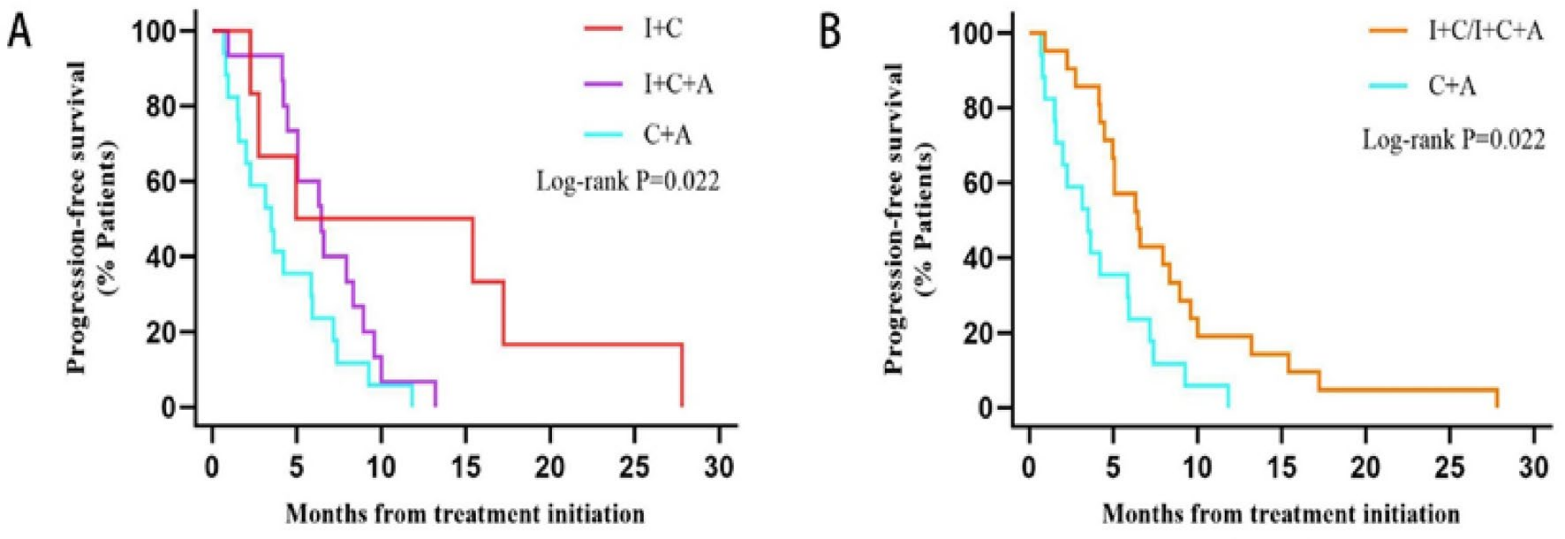
Kaplan–Meier estimates of progression-free survival (PFS) among patients in the brain metastases group: **A** I + C vs I + C + A vs C + A. **B** I + C/I + C + A vs C + A

The statistically significant difference was in PFS for patients with brain metastasis, the median PFS of 21 patients in the I + C and I + C + A groups (6.44 months, 95% CI 4.200–8.680) was obviously higher than that of 17 patients in the C + A group (4.21 months, 95% CI 1.336–7.084). The difference between the two groups was statistically significant (*P* = 0.022). (Fig. 4).

The statistically significant difference was also in PFS for patients with liver metastasis (0.95 vs 6.44 vs 3.48 months, *P* < 0.001). (Supplemental Fig. 1).

### Prognostic factors for PFS

**Fig. 5:**
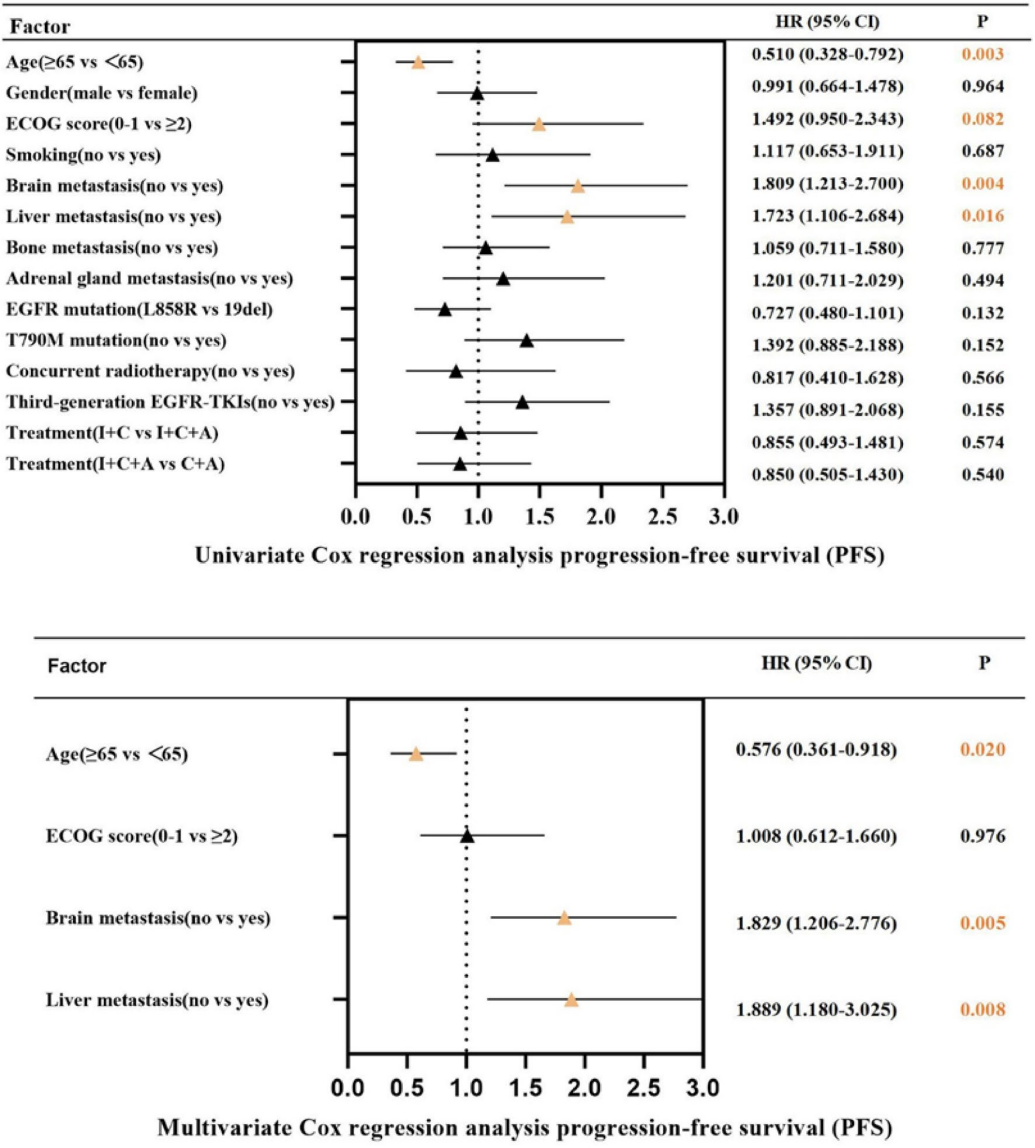
Univariate and multivariate cox regression analysis for progression-free survival (PFS)

**Fig. 6:**
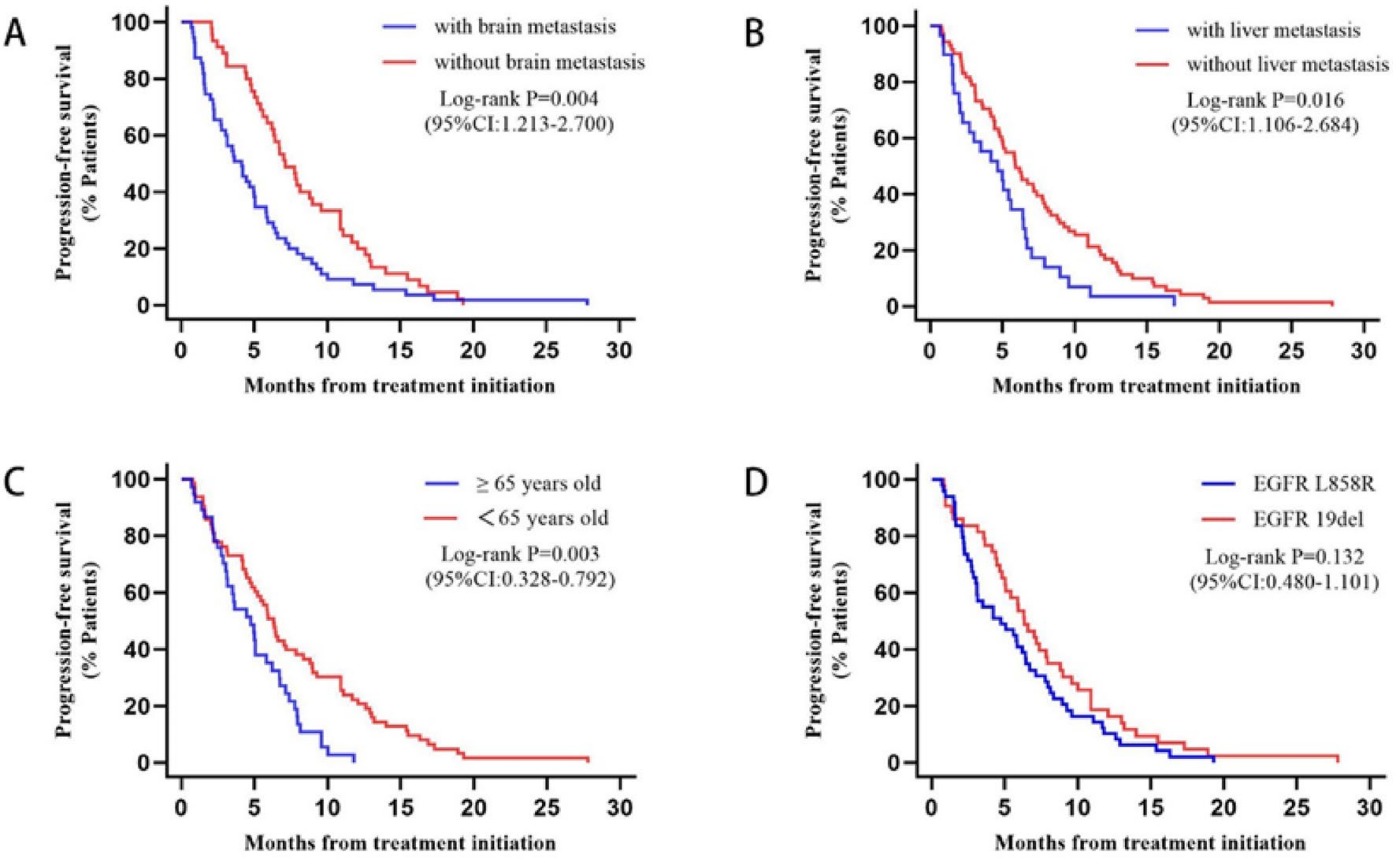
Kaplan–Meier estimates of progression-free survival (PFS) in patients with or without brain metastasis, liver metastasis, ≥ 65 years old vs < 65 years old, and EGFR exon 19 deletion (19del) or EGFR L858R mutation: **A** with or without brain metastasis. **B** with or without liver metastasis. **C** ≥ 65 years old vs < 65 years old. **D** EGFR exon 19 deletion (19del) or EGFR L858R mutation

We further explored the factors affecting overall patient PFS through Cox proportional hazard modeling analysis, and we found that the outcomes of univariate Cox proportional hazard modeling analysis of four variables, namely age, ECOG score, liver metastasis, and brain metastasis, were suggestive of correlation with PFS in all patients. Our multifactorial Cox proportional hazard modeling analysis of these four variables revealed that liver metastasis, brain metastasis, and age were strongly associated with PFS in all patients. Patients with liver metastases and those with brain metastases portended a worse survival prognosis, and patients with EGFR L858R mutation possessed a worse prognosis than those with EGFR 19del (P = 0.132). (Figs. 5, 6) (Supplemental Table 2).

### Adverse events

It has been found that the Grade 3–4 adverse events occurred in 23 of 118 patients, with more adverse events occurring in the C + A group. No treatment-related deaths were detected. (Supplemental Table 3).

## Discussion

This study indicated that although a statistically significant difference was not discovered in PFS among the three groups of advanced NSCLC patients who chose ICIs + chemotherapy, ICIs + chemotherapy + antiangiogenic therapy, and chemotherapy + antiangiogenic therapy after EGFR-TKIs resistance, patients receiving triple therapy are more likely to have better survival benefits. And those patients with brain or liver metastasis can benefit from immune-related therapeutic regimens.

According to numerous previous researches, there is a synergistic antitumor effect between immunotherapy, antiangiogenic therapy, and chemotherapy [16–19]. Based on the mutual synergistic antitumor effects, the Impower150 trial further explored that chemotherapy + immunotherapy + anti-angiogenic therapy as first-line therapy in NSCLC patients with EGFR mutations possessed better clinical efficacy than chemotherapy + immunotherapy and chemotherapy + anti-angiogenic therapy, despite the greater toxicity effects [20]. Furthermore, the Orient-31 trial explored a similar study approach in NSCLC patients after EGFR-TKIs resistance, validating that Sintilimab + the anti-angiogenic therapy biosimilar IBI305 + chemotherapy group had superior PFS and OS in comparison to the chemotherapy group [21]. In terms of the significant findings of the Impower150 and Orient-31 trials, more and more studies are exploring and validating the significance of ICIs in prolonging the survival of patients with advanced NSCLC [23, 24]. However, no retrospective study has yet definitively compared which is better in terms of survival prolongation in NSCLC patients after EGFR-TKIs resistance between immune-related combination therapies and chemotherapy + antiangiogenic therapy which is commonly used in the clinic. In this study, we found that the ORR of such patients applying triple therapy were significantly higher than those of chemotherapy + antiangiogenic therapy, and although the difference in PFS among the three groups was no statistical significance, the Kaplan–Meier curves of the final data analysis indicated that the 12-month PFS was obviously longer in the triple-therapy group, and our results further supported the outcomes of the Impower150 and Orient-31 trials. Furthermore, we consider that the reason leading to the reduced survival advantage in the triple-therapy group may be due to a reduction in therapeutic dosage or prolonged dosing intervals at the later stage because of physical intolerance, which leads to a possible bias in the results.

Brain metastasis has long been the most common type of distant metastasis in NSCLC, and the prognosis of patients is extremely poor. Even though there are several therapeutic approaches available for treating NSCLC patients, the therapeutic efficacy of NSCLC patients with brain metastasis is still unsatisfactory [25]. According to the KEYNOTE-010 trial and the KEYNOTE-042 trial, pembrolizumab revealed better efficacy in advanced NSCLC patients with positive PD-L1 expression than chemotherapy [26, 27]. Pooling the outcomes of 46 NSCLC patients with brain metastases from the CheckMate-063 trial, CheckMate-017 trial, and CheckMate-057 clinical trial found that Nivolumab prevented the progression of brain metastatic lesions [28, 29]. It can be demonstrated that anti-PD-L1-related immunotherapy can bring new hope to NSCLC patients with brain metastases. Considering the possible mechanism is that anti-PD-L1 inhibitors can activate and proliferate T cells in extracranial and lymphoid tissues on the one hand, and on the other hand, several anti-PD-L1 antibodies can directly traverse the blood–brain barrier and promote the activation and proliferation of T cells in intracranial tissues, thus exerting intracranial antitumor activity [30]. Results from pooling the recent analysis of KEYNOTE-021, KEYNOTE-189, and KEYNOTE-407 trials indicated that pembrolizumab combined with chemotherapy results in preferable clinical efficacy than mono-chemotherapy in NSCLC patients with brain metastases [31, 32]. Chemotherapy in combination with Sintilimab, an anti-PD-1 inhibitor approved in China, also observed prolonged PFS in NSCLC patients with brain metastases in comparison to chemotherapy alone [33]. Many researchers have suggested that NSCLC patients with brain metastases can achieve better clinical outcomes with bevacizumab + chemotherapy than with chemotherapy alone [34, 35]. In addition, the Impower150 trial found significant efficacy of chemotherapy + immunotherapy + anti-angiogenic therapy group in patients with liver metastases, but the data on the efficacy of brain metastases were lacking [25]. Although the Orient-31 trial has found that immunotherapy + chemotherapy + antiangiogenic therapy was preferable to mono-chemotherapy in NSCLC patients with brain metastases, it has not compared the efficacy of the above three combination therapies [21]. Besides, Yi Hu et al. [36] showed that advanced NSCLC patients had better PFS and OS after EGFR-TKIs resistance with immune-combination therapy compared to immunologic monotherapy. However, Yi Hu et al. did not evaluate the treatment options for NSCLC patients with brain or liver metastases after resistance to EGFR-TKIs and did not examine the efficacy comparison with chemotherapy + anti-angiogenic therapy which was commonly used in clinical practice. Moreover, XJ Meng et al. evaluated brain metastatic lesions in advanced NSCLC patients after EGFR-TKIs resistance and found that immunotherapy + chemotherapy had effective intracranial efficacy and clearly prolonged patients’ PFS compared with chemotherapy [37]. But the study also only explored the survival differences between different immunotherapies. This retrospective study of ours not only further validates immunotherapy + antiangiogenic therapy + chemotherapy as a therapy that is a relatively better clinical strategy in advanced NSCLC patients after EGFR-TKIs resistance, but also provides the groundbreaking finding that such patients with brain metastases can achieve better efficacy with immune-related combination therapy than with conventional chemotherapy + antiangiogenic therapy, with the difference being statistically significant (median PFS, 6.44 vs 4.21 months, *P* = 0.022). This provides a clinical rationale for preferring immune-combination therapy for NSCLC patients who have brain metastases after EGFR-TKIs resistance. This deserves a larger sample size research to study and validate.

Furthermore, our research also provides a clinical rationale for preferring immunotherapy + antiangiogenic therapy + chemotherapy for such patients with liver metastases. Despite the limited sample size, this result is in accord with the results of the Impower150 trial [25].

In this study, ECOG score, liver metastasis, brain metastasis, and age have been revealed to be in connection with the survival prognosis of NSCLC patients through univariate Cox proportional hazard modeling analysis. The multivariate Cox analysis further confirmed that liver metastasis, brain metastasis, and age were associated with the PFS of patients. In recent years, increasing evidence has supported the differences between EGFR mutation subtypes, finding that patients with the presence of EGFR L858R have a poorer survival prognosis than those with EGFR 19del, not only in EGFR-TKIs targeted therapies [38], but also in immunotherapy [36]. However, in our study, we did not find statistically different survival results between EGFR L858R and EGFR 19del. Larger sample sizes and further research are needed.

There are a few limitations in our research. Firstly, it is a retrospective study with limited clinical data collected, for example, not enough evaluable data on PD-L1 expression, TMB, etc. were collected. Secondly, the limited sample size may lead to biased results. Thirdly, the rarity of cases of immunotherapy + antiangiogenic therapy led us not to contrast the efficacy of this regimen with the three kinds of therapies in this article. Additionally, this study did not assess the OS on account of the high number of post-treatment confounders.

## Conclusion

In conclusion, this retrospective study demonstrates that advanced NSCLC patients after EGFR-TKIs resistance may be able to achieve better clinical outcomes from the regimen of immunotherapy + antiangiogenic therapy + chemotherapy. Patients with brain metastases tended to favor the application of immune-related combination therapies. Patients with liver metastases preferred triple therapy. Age, brain metastases, and liver metastases affect the survival prognosis of patients.

### Supplementary Information

Below is the link to the electronic supplementary material.

Supplementary file1 (PDF 542 kb)

## Data Availability

Data are available on reasonable request. Anonymized individual participant data and study documents can be requested for further research.
