# Supplementary material for: A retrospective real-world study: the efficacy of immune-related combination therapies in advanced non-small cell lung cancer after resistance to EGFR-TKIs
Source: Cancer Immunol Immunother. 2023 Oct 31;72(12):4355–65. doi: 10.1007/s00262-023-03570-9 (PMC10700213; doi:10.1007/s00262-023-03570-9)
Supplement: Supplementary file 1 — Supplementary file1 (PDF 542 kb) [file 262_2023_3570_MOESM1_ESM.pdf]

Supplemental Table 1. **Clinical characteristics of the study population.**

Characteristic	N (%)			
	**All (N=118)**	**I+C (N=25)**	**I+C+A (N=43)**	**C+A (N=50)**
**Age at the start of treatment**				
≥65	45 (38.14)	12 (48.00)	12 (27.91)	21 (42.00)
< 65	73 (61.86)	13 (52.00)	31 (72.09)	29 (58.00)
**Gender**				
Male	57 (48.31)	13 (52.00)	22 (51.16)	22 (44.00)
Female	61 (51.69)	12 (48.00)	21 (48.84)	28 (56.00)
**Smoking history**				
Never	99 (83.90)	21 (84.00)	35 (81.40)	43 (86.00)
Smoked	19 (16.10)	4 (16.00)	8 (18.60)	7 (14.00)
**ECOG score**				
0-1	89 (75.42)	19 (76.00)	29 (67.44)	41 (82.00)
≥2	29 (24.58)	6 (24.00)	14 (32.56)	9 (18.00)
**Pathologic type**				
Adenocarcinoma	111 (94.07)	21 (84.00)	41 (95.35)	49 (98.00)
Squamous carcinoma	5 (4.24)	4(16.00)	0 (0.00)	1 (2.00)
Mixed type	2 (1.69)	0 (0.00)	2 (4.65)	0 (0.00)
**Sites of metastasis**				
Brain	68 (57.63)	13 (52.00)	28 (65.11)	27 (54.00)
Liver	34 (28.81)	4 (16.00)	12 (27.91)	18 (36.00)

Bone	70 (59.32)	13 (52.00)	25 (58.14)	32 (64.00)
Adrenal gland	19 (16.10)	5 (20.00)	8 (18.60)	6 (12.00)
**Primary EGFR mutation**				
EGFR 19del	54 (49.09)	11 (44.00)	26 (60.47)	17 (34.00)
EGFR 21L858R	51 (46.36)	12 (48.00)	12 (27.91)	27 (54.00)
others	5 (4.55)	1 (4.00)	3 (6.98)	1 (2.00)
**Acquired T790M mutation**				
Yes	30 (25.42)	7 (28.00)	11 (25.58)	12 (24.00)
No or unknown	88 (74.58)	18 (72.00)	32 (74.42)	38 (76.00)
**Previous EGFR-TKIs treatment**				
1st/2nd generation TKI	49 (41.53)	14 (56.00)	10 (23.26)	25 (50.00)
1st/2nd →3rd generation TKI	53 (44.91)	8 (32.00)	26 (60.47)	19 (38.00)
3rd generation TKI	16 (13.56)	3 (12.00)	7 (16.27)	6 (12.00)
**Prior lines of therapy**				
≤2	32 (27.12)	2 (8.00)	12 (27.91)	18 (36.00)
>2	86 (72.88)	23 (92.00)	31 (72.09)	32 (64.00)
**Concurrent radiotherapy**				
No	102 (86.44)	22 (88.00)	34 (79.07)	46 (92.00)
Yes	16 (13.56)	3 (12.00)	9 (20.93)	4 (8.00)
**Radiotherapy site**				
lung	7 (43.75)	2 (66.67)	3 (33.33)	2 (50.00)
bone	7 (43.75)	1 (33.33)	4 (44.44)	2 (50.00)

brain	2 (12.50)	0 (0.00)	2 (22.23)	0 (0.00)

ECOG, Eastern Cooperative Oncology Group; EGFR, epidermal growth factor receptor; 19del, exon 19 deletion; 21L858R, exon 21 L858R mutation; TKI, tyrosine kinase inhibitor; I + C, Immunotherapy + chemotherapy combination treatment; I + C + A, Immunotherapy + chemotherapy + antiangiogenic combination treatment; C + A, chemotherapy + antiangiogenic combination treatment.

Supplemental Table 2 . Univariate and Multivariate Cox regression analysis for progression-free survival (PFS).

Univariate Cox regression analysis for progression-free survival (PFS).

**Factor**	**HR**	**95% CI**	**P**
Age(≥65 vs ＜65)	0.510	0.328-0.792	0.003
Gender(male vs female)	0.991	0.664-1.478	0.964
ECOG score(0-1 vs ≥2)	1.492	0.950-2.343	0.082
Smoking(no vs yes)	1.117	0.653-1.911	0.687
Brain metastasis(no vs yes)	1.809	1.213-2.700	0.004
Liver metastasis(no vs yes)	1.723	1.106-2.684	0.016
Bone metastasis(no vs yes)	1.059	0.711-1.580	0.777
Adrenal gland metastasis(no vs yes)	1.201	0.711-2.029	0.494
Primary EGFR mutation(L858R vs 19del)	0.727	0.480-1.101	0.132
Secondary EGFR mutation(without T790M vs with T790M)	1.392	0.885-2.188	0.152
Concurrent radiotherapy(no vs yes)	0.817	0.410-1.628	0.566
Prior third-generation EGFR-TKI therapy(no vs yes)	1.357	0.891-2.068	0.155
Treatment(I+C vs I+C+A vs C+A)	0.855	0.493-1.481	0.574
	0.850	0.505-1.430	0.540

Multivariate Cox regression analysis progression-free survival (PFS)

**Factor**	**HR**	**95% CI**	**P**
Age(≥ 65 vs < 65)	0.576	0.361-0.918	0.020
ECOG score(0-1 vs ≥2)	1.008	0.612-1.660	0.976
Brain metastasis(no vs yes)	1.829	1.206-2.776	0.005
Liver metastasis(no vs yes)	1.889	1.180-3.025	0.008

Supplemental Table 3. Adverse events.

Events	Grade 1-2			Grade 3-4		
	I+C	I+C+A	C+A	I+C	I+C+A	C+A
Leukopenia	7(28.0%)	6(14.0%)	18(36.0%)	2(8.0%)	3(7.0%)	2(4.0%)
Neutropenia	2(8.0%)	4(9.3%)	5(10.0%)	2(8.0%)	2(4.7%)	8(16.0%)
Thrombocytopenia	1(4.0%)	3(7.0%)	7(14.0%)	0(0.0%)	0(0.0%)	1(2.0%)
Anemia	4(16.0%)	2(4.7%)	7(14.0%)	0(0.0%)	1(2.3%)	2(4.0%)
Transaminitis	1(4.0%)	4(9.3%)	8(16.0%)	0(0.0%)	1(2.3%)	0(0.0%)
Fever	2(8.0%)	3(8.7%)	6(12.0%)	0(0.0%)	0(0.0%)	0(0.0%)
Pneumonia	1(4.0%)	1(2.3%)	1(2.0%)	0(0.0%)	0(0.0%)	0(0.0%)
Hypothyroidism	3(12.0%)	0(0.0%)	0(0.0%)	0(0.0%)	0(0.0%)	0(0.0%)
Rash	0(0.0%)	0(0.0%)	0(0.0%)	1(4.0%)	1(2.3%)	0(0.0%)
Vomiting	0(0.0%)	0(0.0%)	1(2.0%)	0(0.0%)	0(0.0%)	0(0.0%)
Anorexia	2(8.0%)	0(0.0%)	0(0.0%)	0(0.0%)	1(2.3%)	0(0.0%)
Limb numbness	0(0.0%)	0(0.0%)	1(2.0%)	0(0.0%)	2(4.7%)	0(0.0%)
Hypopituitarism	0(0.0%)	4(9.3%)	0(0.0%)	0(0.0%)	0(0.0%)	0(0.0%)
Myocarditis	0(0.0%)	0(0.0%)	0(0.0%)	0(0.0%)	0(0.0%)	0(0.0%)
Hepatitis	0(0.0%)	0(0.0%)	0(0.0%)	0(0.0%)	0(0.0%)	0(0.0%)
Proteinuria	0(0.0%)	1(2.3%)	0(0.0%)	0(0.0%)	0(0.0%)	0(0.0%)
Epistaxis	0(0.0%)	1(2.3%)	0(0.0%)	0(0.0%)	0(0.0%)	0(0.0%)
Thrombosis	0(0.0%)	1(2.3%)	0(0.0%)	0(0.0%)	0(0.0%)	0(0.0%)

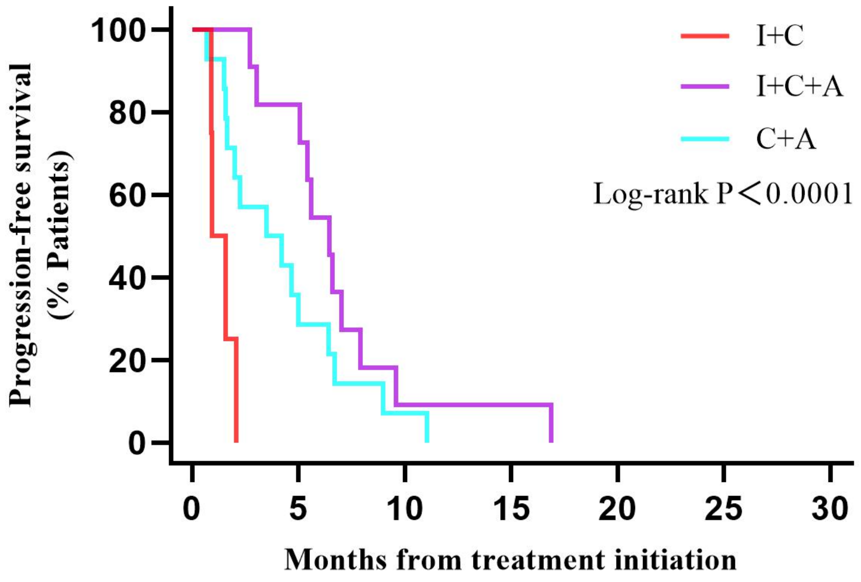

Supplemental Figure 1. Kaplan-Meier estimates of progression-free survival (PFS) among the three groups of patients with liver metastases.

Supplemental Table 4. The specific names and percentages of Immunotherapy, Chemotherapy regimens, EGFR-TKIs, and Antiangiogenic therapy.

Immunotherapy (N %)	Chemotherapy (N %)	EGFR-TKIs (N %)	Antiangiogenic therapy (N %)
Toripalimab (14/20.59%)	pemetrexed+platinum (45/38.14%)	Gefitinib (52/44.07%)	Bevacizumab (76/81.72%)
Pembrolizumab (6/8.82%)	Paclitaxel+platinum (14/11.86%)	Erlotinib (24/20.34%)	Anlotinib (17/18.28%)
Sintilimab (24/35.29%)	Docetaxel+platinum (3/2.54%)	Icotinib (25/21.19%)	
Tislelizumab (7/10.29%)	pemetrexed (28/23.73%)	Afatinib (11/9.32%)	
Camrelizumab (9/13.24%)	Gemcitabine (2/1.69%)	Osimertinib (47/39.83%)	
Atezolizumab (3/4.41%)	Paclitaxel (26/22.03%)	Almonertinib (15/12.71%)	
Nivolumab (2/2.94%)		Furmonertinib (7/5.93%)	
Durvalumab (1/1.47%)			
Penpulimab (2/2.94%)			
